# Risk factors of esophagojejunal anastomotic leakage after total gastrectomy for gastric and Siewert type II/III esophagogastric cancer: a retrospective analysis from a tertiary hospital

**DOI:** 10.3389/fonc.2024.1481278

**Published:** 2024-11-28

**Authors:** Junjie Liu, Jinzhi Hu, Jiaming Fang, Yingliang Chen, Yonghe Chen, Jiasheng He, Zijian Deng, Junsheng Peng, Lei Lian, Shi Chen

**Affiliations:** ^1^ Department of General Surgery (Gastric Surgery), The Sixth Affiliated Hospital, Sun Yat-sen University, Guangzhou, China; ^2^ Guangdong Provincial Key Laboratory of Colorectal and Pelvic Floor Diseases, The Sixth Affiliated Hospital, Sun Yat-sen University, Guangzhou, China; ^3^ Biomedical Innovation Center, The Sixth Affiliated Hospital, Sun Yat-sen University, Guangzhou, China; ^4^ Department of Ultrasound, The Sixth Affiliated Hospital, Sun Yat-sen University, Guangzhou, China

**Keywords:** esophagojejunal anastomotic leakage, laparoscopic surgery, total gastrectomy, gastric cancer, esophagogastric cancer

## Abstract

**Background and objectives:**

To detect the risk factors associated with esophagojejunal anastomotic leakage (EJAL) after total gastrectomy for gastric and Siewert type II/III esophagogastric cancer.

**Methods:**

The data for 609 patients underwent Roux-en-Y esophagojejunostomy after total gastrectomy between March 2015 and March 2021 were reviewed. Univariate and multivariate analyses were performed to evaluate the risk factors.

**Results:**

EJAL was observed in 48 (7.9%) of 609 patients. Univariate analysis revealed that gender, the number of comorbidities (hypertension, diabetes mellitus, coronary heart disease and chronic obstructive pulmonary disease), postoperative serum albumin, tumor location, duration of operation were risk factors associated with EJAL. Patients who had the following factors including male, the number of comorbidities ≥2, postoperative serum albumin <35 g/L, tumor location was esophagogastric junction, duration of operation ≥260 min were more likely to develop EJAL than those who had not. Multivariate analysis revealed that the number of comorbidities (OR 3.464, 95% CI 1.178 – 10.189, *p* = 0.024) and duration of operation (OR 2.657, 95% CI 1.242 – 5.685, *p* = 0.012) were independent risk factors associated with EJAL.

**Conclusions:**

More morbidities and prolonged operative duration were independently associated with EJAL after total gastrectomy for gastric and Siewert type II/III esophagogastric cancer. This study indicated the necessity for careful management of these high-risk patients.

## Introduction

Gastric cancer is the fifth most common malignant tumor in the world and fourth for mortality (1). The incidence of upper gastric cancer is increasing, especially for esophagogastric junction (EGJ) cancer (2, 3). Accordingly, total gastrectomy is carried out more frequently (4, 5). Compared to distal gastrectomy, total gastrectomy (TG) is technically more difficult. Esophagojejunal anastomotic leakage (EJAL) has been considered as one of the most noteworthy complications after total gastrectomy (6). The incidence of EJAL varies between 2.1% and 14.6% (7) and can be lethal in some serious cases. EJAL deteriorates life quality and survival of gastric cancer patients (8, 9). It is important to fulfill safe and satisfied esophagojejunostomy in total gastrectomy. Although several studies have investigated risk factors of EJAL, the widespread use of laparoscopic gastrectomy and increasing incidence of EGJ cancer impelled us to make the present study. Therefore, this study aimed to identify the risk factors of EJAL in gastric and Siewert type II/III esophagogastric cancer patients receiving total gastrectomy.

## Methods

### Patients

Between March 2015 and March 2021, patients who were diagnosed as gastric adenocarcinoma and underwent total gastrectomy in our medical center were included in this study. Esophagogastric junction adenocarcinoma was included except for the Siewert type I tumor.

We obtained approval from the institutional review board for this retrospective study.

### Surgical techniques

The total gastrectomy with D2 lymph nodes dissection was performed according to the Japanese gastric cancer treatment guidelines (fourth edition). In the Siewert type II tumors, total gastrectomy with negative esophageal resection margin was needed. Roux-en-Y reconstruction was routinely used. Linear or circular staplers were both used, which were selected basing on the experiences of surgeons and the actual surgical situations. Laparoscopic surgery of total gastrectomy included peri-gastric isolation, lymph node dissection, and blood vessel ligation. Digestive tract reconstruction could be completed via an abdominal incision not greater than 10 cm or even via laparoscopy. Totally laparoscopic total gastrectomy was defined as esophagojejunal anastomosis was completed via laparoscopy and linear stapler was routinely used. Laparoscopic-assisted total gastrectomy was defined as esophagojejunal anastomosis was completed via abdominal incision not greater than 10 cm and circular stapler was routinely used. For the open total gastrectomy patients, the procedure was similar with that of laparoscopic surgery except it was performed under direct visualization with the length of the auxiliary incision exceeded 10 cm. In this study, "conversion to open" in the laparoscopic surgery was defined as cases requiring laparotomy or auxiliary incision lengths greater than 10 cm.

### Diagnosis of EJAL

Radiological leakage was defined as transudation of the water-soluble contrast medium outside the anastomotic site seen on X-ray imaging, and/or peri-anastomotic fluid, abscess, and free air shown by CT, and/or fistula suggested by endoscopy. Clinical leakage was diagnosed by the presence of turbid fluid or intestinal content or intake chyme from the drainage tube accompanied by fever, or abdominal pain, or elevated inflammation markers such as white blood cell count, or leukocyte count, or C-reactive protein (CRP) or procalcitonin (PCT), and/or defective integrity of the esophagojejunal anastomosis during relaparotomy. Radiological leakage or clinical leakage for esophagojejunal anastomosis was considered EJAL in the present study. When clinical leakage was suspected but without radiological signs, methylene blue swallowed and drainage observation helped to confirm the diagnosis of EJAL. Additionally, the exclusion of other complications such as duodenal stump leakage, jejunojejunostomy leakage, pancreatic fistula, and chylous fistula helped us to confirm the EJAL diagnosis.

### Data collection and variables

The variables were obtained retrospectively from the medical records and surgical records. The variables potentially associated with EJAL were composed of 3 parts: patient-related, tumor-related, and surgery-related variables.

Patient-related variables included age (<65, ≥65 years old), sex, American Society of Anesthesiologists (ASA) category (I–III), smoking, body mass index (BMI) (<25, ≥25 kg/m^2^), presence of hypertension, presence of diabetes, presence of coronary heart disease, presence of chronic obstructive pulmonary disease (COPD), the number of comorbidities (0–1, 2–4), the history of abdominal surgery, preoperative chemotherapy, preoperative hemoglobin (<100 versus ≥100 g/L), preoperative serum albumin (<35, ≥35 g/L), preoperative carcinoembryonic antigen (CEA) (<5, ≥5 ng/mL), postoperative hemoglobin (<90, ≥90 g/L), and postoperative serum albumin (<35, ≥35 g/L). The number of comorbidities was defined as the amount of the four underlying diseases: hypertension, diabetes, coronary heart disease, and chronic obstructive pulmonary disease (COPD).

Tumor-related variables included tumor location (EGJ, not EGJ), tumor differentiation (well, moderate, poor differentiation, other), Lauren type (diffuse, mixed, intestinal), vascular invasion, perineural invasion, the tumor size (maximum length of diameter <5, ≥5 cm), depth of invasion (T0 – T4), lymph node status (N0 – N3), and metastatic status (M0 – M1).Surgery-related variables included duration of operation (<260, ≥260 min), blood loss (<300, ≥300 ml), intraoperative blood transfusion, combined organ resection, R0 resection, surgical method (totally laparoscopic, laparoscopic-assisted, open), anastomotic method (circular, linear stapler), surgeon experience (<10, ≥10 years) and date of surgery (March 2015 – March 2018, March 2018 – March 2021). Surgeon experience is defined as the duration from the time obtaining a senior professional post to the date of surgery.

Additionally, the sensitivity of X-ray imaging, CT, and endoscopy for EJAL diagnosis were evaluated. The treatment for the EJAL in each patient was reviewed. Clavien-Dindo classification system was used to stratify for the severity of EJAL.

### Statistical analysis

Statistical analysis was performed with SPSS version 24.0 (IBM Corp., Armonk, NY, USA). Continuous variables were dichotomized according to the clinical situation or previous literature or using the Youden index in receiver operating characteristic (ROC) curve to calculate the cut-off value. The differences between groups were analyzed using the chi-squared test or Fisher's exact test, and p < 0.05 was considered statistically significant. Variables with p < 0.05 in the univariate analysis entered the multivariate analysis. Logistic regression model in the multivariate analysis was used to investigate the independent risk factors associated with EJAL. Odds ratios (OR) and their 95% confidence intervals (CI) were also provided. A *p* value < 0.05 was statistically significant.

## Results

### Patient characteristics

**Table 1** summarized the demographic and pathological characteristics of included patients. From March 2015 to March 2021, 609 eligible patients with gastric cancer underwent total gastrectomy. There were 433 men and 176 women, with a median age of 62 (range: 20-88) years. Of comorbidities, hypertension was the most common. A total of 357 (58.6%) patients had EGJ adenocarcinoma. Of the pathological stage, T3 tumor was the most frequent.

**Table 1:** Patient characteristics.

Variables	Values
Age, years, median (range)	62 (20-88)
Gender, n (%)	
Male	433 (71.1)
Female	176 (28.9)
BMI, kg/m^2^, median (range)	21.8 (13.1-33.3)
ASA category, n (%)	
I	247 (40.5)
II	339 (55.7)
III	23 (3.8)
Comorbid disease, n (%)	
Hypertension	99 (16.3)
Diabetes mellitus	49 (8.0)
Coronary heart disease	12 (2.0)
COPD	51 (8.4)
Tumor location, n (%)	
EGJ	357 (58.6)
Fundus	26 (4.3)
Body	205 (33.7)
Whole stomach	21 (3.4)
Depth of invasion, n (%)	
pT0	26 (4.3)
pT1	61 (10.0)
pT2	59 (9.7)
pT3	357 (58.6)
pT4	106 (17.4)
Lymph node status, n (%)	
pN0	232 (38.1)
pN1	108 (17.7)
pN2	106 (17.4)
pN3	163 (26.8)

### EJAL incidence and descriptive data of EJAL

**Table 2** showed the detail information of EJAL. Of the 609 eligible patients with gastric cancer underwent total gastrectomy, 48 patients had EJAL. The incidence of EJAL was 7.9%. Twenty-seven patients (56.3%) had EJAL with Clavien-Dindo classification IIIa, and in that situation, abdominal puncture, thoracentesis, and enteral nutrition tube insertion were frequently used. Two patients (4.2%) with EJAL underwent relaparotomy (with Clavien-Dindo classification IIIb). Six patients (12.5%) needed treatment of intensive care unit (ICU). There was one patient who died of multiple organ failures resulting from EJAL. The postoperative hospital stay of these EJAL patients was 24 days (range: 5-70). Most EJAL patients undertaken the examination of X-ray imaging and CT (81.2% for X-ray and 77.1% for CT). The positive rate of X-ray imaging and CT for EJAL diagnosis was 47.9% and 60.4%. We also noticed that 52.1% (25/48) EJAL patients were diagnosed with CT or clinical signs and symptoms. There were 11 patients who were not diagnosed with X-ray imaging alone and could be diagnosed with CT after X-ray imaging. Endoscopy was not routinely used to diagnose EJAL but frequently used for the insertion of the enteral nutrition tube. Nearly a half of patients (47.9%) established enteral nutrition support with feeding tube. The incidence of EJAL before March 2018 was 7.28% and 8.19% for the after. **Table 3** showed the detailed information of EJAL diagnosis in 48 EJAL patients.

**Table 2:** Detail of EJAL.

Variables	Values
Clavien-Dindo classification, n (%)	
I	6 (12.5)
II	6 (12.5)
III	
IIIa	27 (56.3)
IIIb	2 (4.2)
IV	
IVa	4 (8.3)
IVb	2 (4.2)
V	1 (2.1)
Postoperative hospital stays, days, median (range)	24 (5-70)
Examinations of X-ray imaging, n (%)	
Positive sign	23 (47.9)
Negative sign	16 (33.3)
Without X-ray	9 (18.8)
Examinations of CT, n (%)	
Positive sign	29 (60.4)
Negative sign	8 (16.7)
Without CT	11 (22.9)
X-ray without positive sign but CT with positive sign	11 (22.9)
Examinations of endoscopy, n (%)	
Endoscopy with positive sign	9 (18.8)
Endoscopy without positive sign	2 (4.2)
Without endoscopy	37 (77.1)
Treatment via nutrition support route, n (%)	
Enteral nutrition support with feeding tube	23 (47.9)
Without enteral nutrition support	25 (52.1)
The incidence of EJAL, %	
March 2015–March 2018	7.28
March 2018–March 2021	8.19

(n=48).

**Table 3:** The diagnosis of the 48 EJAL patients.

No. of patients (n)	X-ray imaging (fluoroscopy)	CT	Endoscopy
4	+	+	+
1	+	+	–
8	+	+	no
2	–	+	+
0	–	+	–
9	–	+	no
1	no	+	+
0	no	+	–
4	no	+	no
0	+	–	+
1	+	–	–
2	+	–	no
1	+	no	+
0	+	no	–
6	+	no	no
1	–	–	+
0	–	–	–
2	–	–	no
0	–	no	+
0	–	no	0
2	–	no	no
0	no	–	+
0	no	–	–
2	no	–	no
0	no	no	+
0	no	no	–
2	no	no	no

+: positive sign; -: negative sign; no: not examined.

### Univariate analysis and Multivariate analysis of risk factors of EJAL

Univariate analysis and multivariate analysis associated with EJAL were presented in **Tables 4** and **5**. Univariate analysis revealed that gender, the number of comorbidities, postoperative serum albumin, tumor location, duration of operation were risk factors associated with EJAL. Patients who had the following factors including male, the number of comorbidities was ≥2, postoperative serum albumin <35 g/L, tumor location was EGJ, duration of operation ≥260 min were more likely to develop EJAL than those who had not (*p* = 0.023, 0.036, 0.032, 0.016, 0.004 respectively). Multivariate analysis revealed that the number of comorbidities (OR 3.464, 95% CI 1.178 – 10.189, *p* = 0.024) and duration of operation (OR 2.657, 95% CI 1.242 – 5.685, *p* = 0.012) were independent risk factors associated with EJAL.

**Table 4:** Univariate analysis of risk factors for EJAL.

Variables	No leakage, n (%)	Leakage, n (%)	*p* value
Gender			0.023
Male	392 (69.9)	41 (85.4)	
Female	169 (30.1)	7 (14.6)	
Age			0.068
<65	366 (65.2)	25 (52.1)	
≥65	195 (34.8)	23 (47.9)	
ASA category			0.645
I	228 (40.6)	19 (39.6)	
II	313 (55.8)	26 (54.2)	
III	20 (3.6)	3 (6.2)	
Smoking			0.061
No	453 (80.7)	44 (91.7)	
Yes	108 (19.3)	4 (8.3)	
BMI, kg/m^2^			0.281
<25	477 (85.0)	38 (79.2)	
≥25	84 (15.0)	10 (20.8)	
Hypertension			0.371
No	472 (84.1)	38 (79.2)	
Yes	89 (15.9)	10 (20.8)	
Diabetes mellitus			0.083
No	519 (92.5)	41 (85.4)	
Yes	42 (7.5)	7 (14.6)	
Coronary heart disease			0.613
No	549 (97.9)	48 (100)	
Yes	12 (2.1)	0 (0)	
COPD			0.788
No	513 (91.4)	45 (93.8)	
Yes	48 (8.6)	3 (6.2)	
The number of comorbidities			0.036
0–1	539 (96.1)	43 (89.6)	
≥2	22 (3.9)	5 (10.4)	
The history of abdominal surgery			0.163
No	514 (91.6)	47 (97.9)	
Yes	47 (8.4)	1 (2.1)	
Preoperative chemotherapy			0.81
No	388 (69.2)	34 (70.8)	
Yes	173 (30.8)	14 (29.2)	
Preoperative hemoglobin, g/L			0.429
<100	146 (26.0)	10 (20.8)	
≥100	415 (74.0)	38 (79.2)	
Preoperative serum albumin, g/L			0.092
<35	115 (20.5)	5 (10.4)	
≥35	446 (79.5)	43 (89.6)	
Preoperative CEA, ng/mL			0.294
<5	424 (75.6)	33 (68.8)	
≥5	137 (24.4)	15 (31.2)	
Postoperative hemoglobin, g/L			0.50
<90	129 (23.0)	9 (18.8)	
≥90	432 (77.0)	39 (81.2)	
Postoperative serum albumin, g/L			0.032
<35	491 (87.5)	47 (97.9)	
≥35	70 (12.5)	1 (2.1)	
Tumor location			0.016
EGJ	321 (57.2)	36 (75.0)	
Not EGJ	240 (42.8)	12 (25.0)	
Tumor differentiation			0.133
Well	24 (4.3)	30 (62.5)	
Moderate	126 (22.5)	13 (27.1)	
Poor	393 (70.1)	5 (10.4)	
Other	18 (3.2)	0 (0.0)	
Lauren type			0.055
Diffuse	233 (41.5)	14 (29.2)	
Mixed	182 (32.4)	14 (29.2)	
Intestinal	146 (26.0)	20 (41.7)	
Vascular invasion			0.935
No	359 (64.0)	31 (64.6)	
Yes	202 (36.0)	17 (35.4)	
Perineural invasion			0.659
No	297 (52.9)	27 (56.2)	
Yes	264 (47.1)	21 (43.8)	
Tumor size, cm			0.182
<5	355 (63.3)	35 (72.9)	
≥5	206 (36.7)	13 (27.1)	
Depth of invasion			0.904
T0	24 (4.3)	2 (4.2)	
T1	55 (9.8)	6 (12.5)	
T2	53 (9.4)	6 (12.5)	
T3	330 (58.8)	27 (56.2)	
T4	99 (17.6)	7 (14.6)	
Lymph node status			0.917
N0	212 (37.8)	20 (41.7)	
N1	99 (17.6)	9 (18.8)	
N2	98 (17.5)	8 (16.7)	
N3	152 (27.1)	11 (22.9)	
Metastatic status			0.859
M0	507 (90.4)	43 (89.6)	
M1	54 (9.6)	5 (10.4)	
Duration of operation, min			0.004
<260	225 (40.1)	9 (18.8)	
≥260	336 (59.9)	39 (81.2)	
Blood loss, ml			0.779
<300	385 (68.6)	32 (66.7)	
≥300	176 (31.4)	16 (33.3)	
Intraoperative blood transfusion			0.558
No	473 (84.3)	42 (87.5)	
Yes	88 (15.7)	6 (12.5)	
Combined organ resection			0.753
No	477 (85.0)	40 (83.3)	
Yes	84 (15.0)	8 (16.7)	
R0 resection			0.532
Yes	527 (93.9)	44 (91.7)	
No	34 (6.1)	4 (8.3)	
Surgical method			0.142
Totally laparoscopic	148 (26.4)	13 (27.1)	
Laparoscopic assisted	256 (45.6)	29 (60.4)	
Open	157 (28.0)	6 (12.5)	
Anastomotic method			0.736
Circular stapler	410 (73.1)	34 (70.8)	
Linear stapler	151 (26.9)	14 (29.2)	
Date of surgery			0.694
March 2015 – March 2018	191 (34.0)	15 (31.2)	
March 2018 – March 2021	370 (66.0)	33 (68.8)	
Surgeon experience, years			0.179
<10	302 (53.8)	21 (43.8)	
≥10	259 (46.2)	27 (56.2)	

**Table 5:** Multivariate analysis of risk factors for EJAL.

Variables	OR	95% CI	*p* value
Gender			
Male	1		
Female	0.511	0.218–1.196	0.122
The number of comorbidities			
0–1	1		
≥2	3.464		
1.178–10.189	0.024		
Postoperative serum albumin, g/L			
<35	1		
≥35	0.177	0.024–1.332	0.093
Tumor location			
EGJ	1		
Not EGJ	0.583	0.289–1.175	0.131
Duration of operation, min			
<260	1		
≥260	2.657	1.242–5.685	0.012

### Univariate analysis and Multivariate analysis of associated factors for prolonged duration of operation

Univariate analysis and multivariate analysis associated with prolonged duration of operation were presented in **Tables 6** and **7**. Univariate analysis revealed that receiving preoperative chemotherapy, blood loss ≥300 ml in surgery, the need for intraoperative blood transfusion, laparoscopic surgery, the date of surgery before March 2018, and the surgeon experience <10 years were associated with prolonged duration of operation. Multivariate analysis revealed that receiving preoperative chemotherapy (OR 2.000, 95% CI 1.323 – 3.023, *p* = 0.001), intraoperative blood transfusion (OR 2.166, 95% CI 1.201 – 3.871, *p* = 0.008), laparoscopic surgery (OR 7.544, 95% CI 4.921 – 11.567, *p* < 0.0001) and surgeon experience (OR 0.6, 95% CI 0.362 – 0.994, *p* = 0.047) were independently associated with prolonged duration of operation.

**Table 6:** Univariate analysis of associated factors for prolonged duration of operation.

Variables	Operative duration <260 min, n (%)	Operative duration ≥260 min, n (%)	*p* value
Gender			0.057
Male	156 (66.7)	277 (73.9)	
Female	78 (33.3)	98 (26.1)	
Age, years			0.177
<65	158 (67.5)	233 (62.1)	
≥65	76 (32.5)	142 (37.9)	
ASA category			0.154
I	97 (41.5)	150 (40.0)	
II	124 (53.0)	215 (57.3)	
III	13 (5.6)	10 (2.7)	
BMI, kg/m^2^			0.06
<25	217 (92.7)	330 (88.0)	
≥25	17 (7.3)	45 (12.0)	
Hypertension			0.255
No	201 (85.9)	309 (82.4)	
Yes	33 (14.1)	66 (17.6)	
Diabetes mellitus			
No	219 (93.6)	341 (90.9)	0.241
Yes	15 (6.4)	34 (9.1)	
Coronary heart disease			0.816
No	229 (97.9)	368 (98.1)	
Yes	5 (2.1)	7 (1.9)	
COPD			0.435
No	217 (92.7)	341 (90.9)	
Yes	17 (7.3)	34 (9.1)	
The history of abdominal surgery			0.655
No	217 (92.7)	344 (91.7)	
Yes	17 (7.3)	31 (8.3)	
Preoperative chemotherapy			<0.0001
No	183 (78.2)	239 (63.7)	
Yes	51 (21.8)	136 (36.3)	
Preoperative serum albumin, g/L			0.306
<35	51 (21.8)	69 (18.4)	
≥35	183 (78.2)	306 (81.6)	
Tumor location			0.085
EGJ	127 (54.3)	230 (61.3)	
Not EGJ	107 (45.7)	145 (38.7)	
Tumor differentiation			0.533
Well	14 (6.0)	15 (4.0)	
Moderate	55 (23.5)	84 (22.4)	
Poor	160 (68.4)	263 (70.1)	
Other	5 (2.1)	13 (3.5)	
Lauren type			0.089
Diffuse	103 (44.0)	144 (38.4)	
Mixed	63 (26.9)	133 (35.5)	
Intestinal	68 (29.1)	98 (26.1)	
Vascular invasion			0.06
No	139 (59.4)	251 (66.9)	
Yes	95 (40.6)	124 (33.1)	
Perineural invasion			0.055
No	113 (48.3)	211 (56.3)	
Yes	121 (51.7)	164 (43.7)	
Tumor size, cm			0.98
<5	150 (64.1)	240 (64.0)	
≥5	84 (35.9)	135 (36.0)	
Depth of invasion			0.662
T0	7 (3.0)	19 (5.1)	
T1	23 (9.8)	38 (10.1)	
T2	21 (9.0)	38 (10.1)	
T3	138 (59.0)	219 (58.4)	
T4	45 (19.2)	61 (16.3)	
Lymph node status			0.057
N0	78 (33.3)	154 (41.1)	
N1	36 (15.4)	72 (19.2)	
N2	44 (18.8)	62 (16.5)	
N3	76 (32.5)	87 (23.2)	
Metastatic status			0.452
M0	214 (91.5)	336 (89.6)	
M1	20 (8.5)	39 (10.4)	
Blood loss, ml			0.004
<300	212 (90.6)	308 (82.1)	
≥300	22 (9.4)	67 (17.9)	
Intraoperative blood transfusion			0.005
No	210 (89.7)	305 (81.3)	
Yes	24 (10.3)	70 (18.7)	
Combined organ resection			0.436
No	202 (86.3)	315 (84.0)	
Yes	32 (13.7)	60 (16.0)	
R0 resection			0.836
Yes	220 (94.9)	351 (93.6)	
No	14 (6.0)	24 (6.4)	
Laparoscopic surgery			<0.0001
No	119 (50.9)	60 (16.0)	
Yes	115 (49.1)	315 (84.0)	
Date of surgery			0.021
March 2015 – March 2018	66 (28.2)	140 (37.3)	
March 2018 – March 2021	168 (71.8)	235 (62.7)	
Surgeon experience, years			0.019
<10	110 (47.0)	213 (56.8)	
≥10	124 (53.0)	162 (43.2)	

**Table 7:** Multivariate analysis of associated factors for prolonged duration of operation.

Variables	OR	95% CI	*p* value
Preoperative chemotherapy			
No	1		
Yes	2.000	1.323–3.023	0.001
Blood loss, ml			
<300	1		
≥300	1.531	0.996–2.353	0.052
Intraoperative blood transfusion			
No	1		
Yes	2.166	1.201–3.871	0.008
Laparoscopic surgery			
No	1		
Yes	7.544	4.921–11.567	<0.0001
Date of surgery			
March 2015 – March 2018	1		
March 2018 – March 2021	0.663	0.385–1.143	0.139
Surgeon experience, years			
<10	1		
≥10	0.6	0.362–0.994	0.047

## Discussion

EJAL is considered to be one of the most serious complications after total gastrectomy. It results in prolonged hospital stays and increased mortality (10). It is important to modify risk factors during the perioperative period of total gastrectomy (11). The present study demonstrated that EJAL after total gastrectomy for gastric and Siewert type II/III esophagogastric cancer was independently associated with comorbidities and prolonged duration of operation. Therefore, the proper perioperative management specific to these high-risk patients could reduce the presence of postoperative EJAL.

The present of study showed the incidence of EJAL was 7.9%, which was higher than some previous studies (12–14). We analyzed cases from the early phase of introduction of laparoscopic surgery of TG, which might explain that the high incidence of EJAL. Moreover, more than half patients were diagnosed with EGJ cancers. The incidence of EJAL in EGJ cancer patients is generally higher than other upper gastric cancer. We assumed that the rate of EJAL may be underestimate by clinicians because of the over-relying on the X-ray imaging with water-soluble contrast swallowed. A study showed that the contrast-enhanced swallow examination had limited diagnostic efficiency for detecting EJAL, with a sensitivity of 66% (15). In our study, the positive rate of X-ray for EJAL diagnosis was only 47.9% which is lower than that of CT (60.4%). CT displayed the information of fluid or abscess in the thoracic or abdominal cavity, the inflammation reaction of the anastomotic site and the other organs. In our study, 11 patients can't be diagnosed with X-ray imaging alone and could be diagnosed with CT immediately after X-ray imaging. There were 25 patients who were not diagnosed with the X-ray imaging (negative sign or not examined) but diagnosed with CT or other clinical symptoms or sign. For this reason, we strongly recommended CT examinations in the EJAL suspected cases. CT scans after oral contrast helped to diagnose EJAL better.

The safety and feasibility of laparoscopic distal gastrectomy has been proved by several high-quality studies. However, there are still limited evidence in terms of the feasibility and efficacy of laparoscopic TG for gastric cancer patients. The CLASS 02 and KLASS 03 multicenter randomized clinical trials showed that laparoscopic TG was feasible and safe in clinical stage I gastric cancer (16, 17). The laparoscopic TG included laparoscopy-assisted and total laparoscopic surgery (laparoscopic anastomosis). Whether or not laparoscopic TG can increase the risk of EJAL is still on debate. Some studies reported a significant increase in the incidence of EJAL in the laparoscopic TG (13, 18, 19). Totally laparoscopic TG remains a big challenge especially for the unexperienced surgeons. Anastomosis is often performed in the narrow esophageal hiatus for the esophagogastric cancer. Laparoscopic anastomosis with linear stapling has better vision, compared with open anastomosis. In the present study, surgical methods (Totally laparoscopic or laparoscopic assisted or open) and anastomotic methods (circular or linear stapler) did not significantly influence the risk of EJAL. It indicated that no matter which anastomotic method was used, a meticulous surgery performed by an experienced surgeon was important.

Univariate analysis showed that gender, postoperative serum albumin, and tumor location were associated with EJAL. As far as we know, no previous studies indicated a significant correlation between gender and EJAL. Xing et al. found that all 10 patients who developed EJAL were men, but this was not statistically significant (14). In our study, male patients were more likely to develop EJAL although it was not an independent risk factor. Similar phenomenon was found in the study of AL after colorectal cancer surgery (20, 21). The reason why male gender tended to have higher risk of EJAL was unclear. One study showed that gastric cancer patients with a low prognostic nutritional index (PNI) have a higher risk for EJAL in laparoscopic-assisted total gastrectomy (10). Lower postoperative serum albumin can negatively affect the anastomotic healing. The importance of postoperative nutritional support in improving postoperative outcomes of cancer patients is well recognized. There were few reports about the correlation between tumor location and EJAL. We found that the EGJ cancer patients were more likely to develop EJAL. EGJ cancer is a special tumor type with greater surgical difficulty on total gastrectomy than another upper gastric cancer. The anastomosis in Siewert type II/III cancer patients is not easy for an un-experienced surgeon. The narrow space surrounding esophageal hiatus increases anastomotic difficulty.

It's worth noting that the morbidities in the present study only referred to the four following comorbidities: hypertension, diabetes mellitus, coronary heart disease and COPD. The reason why we chose the above diseases for analysis was that these comorbidities were commonly confronted and potentially related to AL according to clinical practice and literature. Hypertension and coronary heart disease can reflect the unhealthy status of blood vessel and microcirculation. As we known, one crucial point of anastomosis is the blood supply. Diabetes mellitus may make adverse effect on wound or tissue healing. Migita et al. reported that patients with HbA1c ≥7.0% was independently associated with higher rate of EJAL (22). COPD may result in hypoxia which is not benefit for the healing of anastomosis. Some studies reported that impaired respiratory function was associated with risk of EJAL (19, 23). Schietroma et al. found that the risk of EJAL was 49% lower in patients receiving 80% FiO2 than in those receiving 30% FiO2 during and 6 hours after open total gastrectomy (relative risk (RR): 0.61; 95% CI: 0.40 – 0.95) (24). Our data showed the morbidities number ≥2 is an independent risk factor of EJAL. This result revealed that we should attach great importance to these patients. Early and aggressive management for these morbidities may reduce the risk of EJAL. We recommended strict management of blood pressure and blood glucose in perioperative period. For coronary heart disease patients, anticoagulant therapy with low molecular weight heparin were commonly used. Exercise of respiratory function and appropriate oxygen supply could alleviate the harmful effect by COPD.

This study indicated that prolonged operative duration was associated with the occurrence of EJAL. Some studies reported that prolonged duration of operation was related to anastomotic leakage (22–25). The present study showed the similar result. Prolonged operative duration may result from many factors: combined resection of other organs, occurrence of adhesions, more technically complicated tumor cases, poor tissue condition of the patient, more blood loss, difficulty during isolation and anastomosis, and surgeon experience. This study showed that receiving preoperative chemotherapy, the need for intraoperative blood transfusion during surgery, laparoscopic surgery and surgeon's experience less than 10 years contributed to prolonged operative duration. The tumors for patients who need to receive preoperative chemotherapy are generally more advanced. Preoperative chemotherapy may increase difficulty of surgery and risk of hemorrhage because of poorer tissue condition. The need for intraoperative blood transfusion during surgery indicated obvious blood loss in surgery, resulting in prolonged hemostasis time. The operative duration of laparoscopic surgery is generally longer than open surgery because of higher skill requirement for laparoscopic isolation or anastomosis. Surgeons need to get through the learning curve of laparoscopic surgery of total gastrectomy. It is also easy to understand that surgeon experience with more years resulted in less operative time. Therefore, we recommend proper albumin and nutrition supplement for the patients who received preoperative chemotherapy or with high-risk of blood loss. However, patient-related or tumor-related factors associated for prolonged duration of operation were difficult to be changed by surgeons in many cases. Surgeons can make effort on the improvement of surgical skills through accumulating experiences of total gastrectomy in order to reduce the duration of operation and the risk of EJAL. Furthermore, clinicians should pay more attention to the patients with obviously prolonged duration of operation. Strict management and careful monitoring for these patients may help clinicians to detect EJAL in early phase.

This study has several limitations. First, this was a retrospective study which may lead to selection and sampling bias. Second, we didn't obtain the data of the time taken for dissection and anastomosis respectively. It was hard to assume whether shortening the time for dissection or the time for anastomosis could reduce EJAL. Third, we couldn't obtain the data of intraoperative anastomotic problems from the existing records, which may lead to prolonged operative duration.

## Conclusions

In conclusion, this study demonstrated that more morbidities and prolonged operative duration were independently associated with esophagojejunal anastomotic leakage after total gastrectomy for gastric and Siewert type II/III esophagogastric cancer. This study indicated the necessity for careful management of these high-risk patients.

## Data Availability

The original contributions presented in the study are included in the article/**Supplementary Material**. Further inquiries can be directed to the corresponding author.
