# Supplementary material for: Risk factors of esophagojejunal anastomotic leakage after total gastrectomy for gastric and Siewert type II/III esophagogastric cancer: a retrospective analysis from a tertiary hospital
Source: Front Oncol. 2024 Nov 28;14:1481278. doi: 10.3389/fonc.2024.1481278 (PMC11634689; doi:10.3389/fonc.2024.1481278)
Supplement: Supplementary file 1 [file Table1.docx]

**Supplementary Table 1.** Univariate analysis of risk factor for EJAL with Clavien-Dindo classification ≥ III.

| Variables | No EJAL with Clavien-Dindo classification ≥ III , n (%) | EJAL with Clavien-Dindo classification ≥ III , n (%) | *p* value |
| --- | --- | --- | --- |
| Gender  Male  Female  Age, years  ＜65  ≥65  ASA category  I  II  III  Smoking  No  Yes  BMI, kg/m^2^  ＜25  ≥25  Hypertension  No  Yes  Diabetes mellitus  No  Yes  Coronary heart disease  No  Yes  COPD  No  Yes  The number of comorbidities  0–1  ≥2  The history of abdominal surgery  No  Yes  Preoperative chemotherapy  No  Yes  Preoperative hemoglobin, g/L  ＜100  ≥100  Preoperative serum albumin, g/L  ＜35  ≥35  Preoperative CEA, ng/mL  ＜5  ≥5  Postoperative hemoglobin, g/L  ＜90  ≥90  Postoperative serum albumin, g/L  ＜35  ≥35  Tumor location  EGJ  Not EGJ  Tumor differentiation  Well  Moderate  Poor  Other  Lauren type  Diffuse  Mixed  Intestinal  Vascular invasion  No  Yes  Perineural invasion  No  Yes  Tumor size, cm  ＜5  ≥5  Depth of invasion  T0  T1  T2  T3  T4  Lymph node status  N0  N1  N2  N3  Metastatic status  M0  M1  Duration of operation, min  ＜260  ≥260  Blood loss, ml  ＜300  ≥300  Intraoperative blood transfusion  No  Yes  Combined organ resection  No  Yes  R0 resection  Yes  No  Surgical method  Totally laparoscopic  Laparoscopic assisted  Open  Anastomotic method  Circular staler  Linear stapler  Date of surgery  March 2015 – March 2018  March 2018 – March 2021  Surgeon experience, years  ＜10  ≥10 | 401 (70.0)  172 (30.0)  374 (65.3)  199 (34.7)  233 (40.7)  319 (55.7)  21 (3.7)  465 (81.2)  108 (18.8)  514 (89.7)  59 (10.3)  480 (83.8)  93 (16.2)  528 (92.1)  45 (7.9)  561 (97.9)  12 (2.1)  524 (91.4)  49 (8.6)  548 (95.6)  25 (4.4)  526 (91.8)  47 (8.2)  396 (69.1)  177 (30.9)  150 (26.2)  423 (73.8)  117 (20.4)  456 (79.6)  432 (75.4)  141 (24.6)  132 (23.0)  441 (77.0)  502 (87.6)  71 (12.4)  327 (57.1)  246 (42.9)  24 (4.2)  131 (22.9)  400 (69.8)  18 (3.2)  236 (41.2)  186 (32.5)  151 (26.4)  366 (63.9)  207 (36.1)  303 (52.9)  270 (47.1)  365 (63.7)  208 (36.3)  24 (4.2)  55 (9.6)  55 (9.6)  338 (59.0)  101 (17.6)  216 (37.7)  101 (17.6)  101 (17.6)  155 (27.1)  517 (90.2)  56 (9.8)  229 (40.0)  344 (60.0)  391 (68.2)  182 (31.8)  482 (84.1)  91 (15.9)  488 (85.2)  85 (14.8)  538 (93.9)  35 (6.1)    159 (27.7)  264 (46.1)  150 (26.2)  419 (73.1)  154 (26.9)  195 (34.0)  378 (66.0)  303 (52.9)  270 (47.1) | 32 (88.9)  4 (11.1)  17 (47.2)  19 (52.8)  14 (38.9)  20 (55.6)  2 (5.6)  32 (88.9)  4 (11.1)  33 (91.7)  3 (8.3)  30 (83.3)  6 (16.7)  32 (88.9)  4 (11.1)  36 (100)  0 (0)  34 (94.4)  2 (5.6)  34 (94.4)  2 (5.6)  35 (97.2)  1 (2.8)  26 (72.2)  10 (27.8)  6 (16.7)  30 (83.3)  3 (8.3)  33 (91.7)  25 (69.4)  11 (30.6)  6 (16.7)  39 (81.2)  36 (100.0)  0 (0.0)  30 (83.3)  6 (16.7)  5 (13.9)  8 (22.2)  23 (63.9)  0 (0.0)  11 (30.6)  10 (27.8)  15 (41.7)  24 (66.7)  12 (33.3)  21 (58.3)  15 (41.7)  25 (69.4)  11 (30.6)  2 (5.6)  6 (16.7)  4 (11.1)  19 (52.8)  5 (13.9)  16 (44.4)  7 (19.4)  5 (13.9)  8 (22.2)  33 (91.7)  3 (8.3)  5 (13.9)  31 (86.1)  26 (72.2)  10 (27.8)  33 (91.7)  3 (8.3)  29 (80.6)  7 (19.4)  33 (91.7)  3 (8.3)  4 (11.1)  21 (58.3)  11 (30.6)  25 (69.4)  11(30.6)  11 (30.6)  25 (69.4)  20 (55.6)  16 (44.4) | 0.015  0.028 |
|  |  |  | 0.841 |
|  |  |  | 0.373 |
|  |  |  | 0.706 |
|  |  |  | 0.945  0.486 |
|  |  |  | 1.000  0.529 |
|  |  |  |  |
|  |  |  | 0.670 |
|  |  |  |  |
|  |  |  | 0.348 |
|  |  |  | 0.695  0.205  0.085  0.424  0.376  0.015  0.002  0.086  0.129  0.735  0.525  0.486  0.660  0.793  1.000  0.002  0.619  0.339  0.454  0.485  0.089  0.546  0.669 |
|  |  |  | 0.755 |
