# Supplementary material for: Risk factors of esophagojejunal anastomotic leakage after total gastrectomy for gastric and Siewert type II/III esophagogastric cancer: a retrospective analysis from a tertiary hospital
Source: Front Oncol. 2024 Nov 28;14:1481278. doi: 10.3389/fonc.2024.1481278 (PMC11634689; doi:10.3389/fonc.2024.1481278)
Supplement: Supplementary file 2 [file Table2.docx]

**Supplementary table 2.** Multivariate analysis of risk factors for EJAL with Clavien-Dindo classification ≥ III.

| Variables | OR | 95% CI | *p* value |
| --- | --- | --- | --- |
| Gender  Male  Female  Age, years  ＜65  ≥65  Postoperative serum albumin, g/L  ＜35  ≥35  Duration of operation, min  ＜260  ≥260 | 1  0.356  1  1.700  1  ＜0.001  1  3.495 | 0.123–1.046–  0.849–3.396  –  1.329–9.189 | 0.060  0.134  0.997  0.011 |
